# Factors influencing utilisation of maternal health services by adolescent mothers in Low-and middle-income countries: a systematic review

**DOI:** 10.1186/s12884-017-1246-3

**Published:** 2017-02-16

**Authors:** Oluwasola Eniola Banke-Thomas, Aduragbemi Oluwabusayo Banke-Thomas, Charles Anawo Ameh

**Affiliations:** 1 Department of International Public Health, Centre for Maternal and Newborn Health, Liverpool School of Tropical Medicine, Liverpool, UK; 2 McCain Institute for International Leadership, Arizona State University, Tempe, AZ USA

**Keywords:** Adolescents, Maternal health, Utilisation of health services, Maternal health services, Ante-natal care, Delivery, Intra-partum care, Post-natal care

## Abstract

**Background:**

Adolescent mothers aged 15–19 years are known to have greater risks of maternal morbidity and mortality compared with women aged 20–24 years, mostly due to their unique biological, sociological and economic status. Nowhere Is the burden of disease greater than in low-and middle-income countries (LMICs). Understanding factors that influence adolescent utilisation of essential maternal health services (MHS) would be critical in improving their outcomes.

**Methods:**

We systematically reviewed the literature for articles published until December 2015 to understand how adolescent MHS utilisation has been assessed in LMICs and factors affecting service utilisation by adolescent mothers. Following data extraction, we reported on the geographical distribution and characteristics of the included studies and used thematic summaries to summarise our key findings across three key themes: factors affecting MHS utilisation considered by researcher(s), factors assessed as statistically significant, and other findings on MHS utilisation.

**Results:**

Our findings show that there has been minimal research in this study area. 14 studies, adjudged as medium to high quality met our inclusion criteria. Studies have been published in many LMICs, with the first published in 2006. Thirteen studies used secondary data for assessment, data which was more than 5 years old at time of analysis. Ten studies included only married adolescent mothers.

While factors such as wealth quintile, media exposure and rural/urban residence were commonly adjudged as significant, education of the adolescent mother and her partner were the commonest significant factors that influenced MHS utilisation. Use of antenatal care also predicted use of skilled birth attendance and use of both predicted use of postnatal care. However, there may be some context-specific factors that need to be considered.

**Conclusions:**

Our findings strengthen the need to lay emphasis on improving girl child education and removing financial barriers to their access to MHS. Opportunities that have adolescents engaging with health providers also need to be seized. These will be critical in improving adolescent MHS utilisation. However, policy and programmatic choices need to be based on recent, relevant and robust datasets. Innovative approaches that leverage new media to generate context-specific dis-aggregated data may provide a way forward.

**Electronic supplementary material:**

The online version of this article (doi:10.1186/s12884-017-1246-3) contains supplementary material, which is available to authorized users.

## Background

Eighteen Percent of the world population are adolescents, defined as individuals aged 10–19 years [1, 2]. Generally, the global discourse lays emphasis on adolescents aged 15–19 years as they fall within the broader reproductive age group (15–49 years) [3]. About 16 million girls within this 15–19 age group give birth every year, of which 95% of the births occur in low-and middle-income countries (LMICs) [4]. Girls aged 15–19 years contribute to 12% of global annual births however also make up 10% of global annual maternal deaths [4, 5]. Globally, complications during pregnancy and childbirth are the second leading cause of death amongst girls aged 15–19 years old [6]. Recent estimates from 144 countries suggests that adolescents between 15 and 19 years are about one and a half times more likely to die during childbirth when compared with women aged between 20 and 24 years [6], who are relatively better physiologically prepared for pregnancy and childbirth [7]. Ninety-nine percent of these adolescent maternal deaths occur in LMICs (82% occurring in just 20 countries) [6]. About three million girls within this age group undergo unsafe abortions every year, further contributing to these adolescent maternal deaths [8]. For those who survive pregnancy, evidence shows that they have higher risks for postpartum bleeding [9], anaemia, pre-eclampsia and other problems of pregnancy [10, 11]. They also have a higher risk of developing obstetric fistula [12].

Adolescent mothers are not only challenged by the physical threats to their health, as described above, but are also often socially disadvantaged. Many have to raise their babies as single parents, are unable to complete their education and consequently have a limited capacity to secure a job and sustain a livelihood to support themselves and their children [8]. Adolescent mothers have to deal with all these issues while still going through ‘adolescence’ with all its challenges as well as adapting to the maternal role concurrently [13–15].

Furthermore, the health of babies born to adolescent mothers is also at risk as these babies are more prone to preterm delivery, low-birth-weight and of dying as infants compared to those born to 20–24 year-old mothers [9, 16, 17]. Particularly in LMICs, babies born to adolescent mothers face a 50% higher risk of being still born or dying in their first few weeks of life when compared to babies born to mothers between ages 20 and 29 years [8].

These vulnerabilities have been highlighted more recently in the development of the post-2015 agenda, as advocacy for more focus on health of adolescent girls, who have been described as being “left behind” in the era of the Millennium Development Goals (MDGs) has increased [18]. It is well established that utilisation of maternal health services (MHS) across the continuum of care, that is, antenatal, intra-partum (by skilled birth attendants) and postpartum care are critical in reducing pregnancy-related morbidities, decreasing maternal mortality of adolescent mothers and improving outcomes, survival, quality of life and health of their babies [19]. We argue that to better fulfil the promise of the sustainable development goals (SDGs) for adolescent girls during the post-2015 era, strategies that focus on preventing early marriage and early childbearing [20] must be complemented by more research that aims to better understand MHS utilisation patterns of adolescent who become pregnant. Such research would be critical in ensuring that the service needs of this vulnerable group are met.

Therefore, we conducted this systematic review of the literature to explore factors that have been found to influence adolescent utilisation of these life-saving MHS across LMICs, where the burden is greatest. Key questions that we aimed to answer were: How has MHS utilisation by adolescent mothers been assessed? And what are the factors affecting utilisation of MHS by adolescent mothers?

## Methods

We used the PRISMA approach [21] to report findings of this systematic review on factors influencing utilisation of MHS by adolescent mothers in LMICs [22].

### Search strategy

A preliminary search was conducted on Google Scholar® to test the sensitivity of preliminarily identified search terms and to explore other potential search terms that could subsequently be used to identify relevant papers for the review. Following this, we searched through pre-selected databases for relevant peer-reviewed papers. We limited our search to peer-review, as we were interested in finding papers that tested associations of factors through logistic regression. These kinds of papers are almost entirely found in the peer-review literature. In addition, we have focused on the peer-review literature as it guarantees quality checks have been performed before publication.

PubMed, Scopus, Global Health and CINAHL Plus databases were searched. These databases were chosen for their completeness in health-related research areas. The search was limited to papers published in English language. No limit was placed on the start date. However, the search was closed on 31st December 2015 to allow us proceed with the analysis.

Key terms were searched for across the different databases. These terms were grouped into three broad categories.

- Terms which described the group of persons involved: “adolescent mother*”, “teenage mother*”, “adolescent”, “teenager”, “young mother*’, “adolescent pregnan*”, “teenage pregnan*”
- Terms that described type of services used: “maternal health”, “antenatal care”, “prenatal care”, “postnatal care”, “skilled birth attendan*, “delivery”, “obstetric care”
- The single word to link the first two groups: “utilisation”

These terms were combined using Boolean operators in this format ‘(person) AND (service) AND (utilisation)’. Duplicates from the results retrieved from all databases were identified and removed.

Further review of reference lists of the retrieved articles was done to identify any other relevant additional articles that may have been missed in the automated search. In cases when the full-text of the articles could not be retrieved, the author(s) were contacted via the professional social media platform, ResearchGate™ (https://www.researchgate.net/).

The search was independently conducted by two reviewers (SBT and ABT). All three authors (SBT, ABT and CA) reviewed all the records that were retrieved and subsequently agreed on the final eligibility of the retrieved papers based on agreed inclusion and exclusion criteria.

### Inclusion and exclusion criteria

Papers were included if they identified factors affecting utilisation of MHS (antenatal or delivery or postnatal or a combination of any), specifically amongst adolescent mothers (aged between 15 and 19 years) [23] or highlighted adolescent mothers, as part of a wider study. Studies had to be conducted in LMICs (as categorized by the World Bank) [24] and published in English language. Studies that used quantitative or qualitative research, using primary or secondary data and reported the analysis of the data were included for review.

Articles that were commentaries, editorials, non-systematic reviews were excluded from the review.

### Data extraction and synthesis

Upon retrieval, all included papers were allotted unique identifiers for audit purposes. The full texts of the included papers were reviewed, and data was extracted into a pre-developed summary table. This data extraction sheet was developed by all authors during a brainstorming session, leveraging insights from a previously published similar systematic review [25], ensuring that it will sufficiently capture data/information required to answer the review questions.

Data on the author(s), year of publication, the country in which the study was carried out, data source, study subjects, maternal health service(s) (antenatal, delivery and postnatal) studied, study design, analytic framework and sample size were collected. This data framed key descriptive characteristics of the studies relevant for the review and helped to answer our first review question “How has MHS utilisation by adolescent mothers been assessed?” We reported on the geographical distribution of studies that explored adolescent utilisation of MHS in LMICs and summarized characteristics of these studies.

We then collected data on factors considered/predictor variables analysed, statistically significant predictor variables, the strength of association and other results/findings of the analysis to answer our second review question “what are the factors affecting utilisation of MHS by adolescent mothers?” To synthesise the findings from the included studies in response to this question, we used thematic summaries, which allow us to capture of similarities and any variations across the different studies that were included in our review [26, 27]. To achieve this, we present our findings under three predefined themes: Factors considered by researchers in assessing adolescent MHS utilisation, factors assessed as statistically significant, and other findings on MHS utilisation reported in the literature.

### Quality assessment

We used the International Society for Pharmaco-economics and Outcomes Research (ISPOR) Good Research Practices for Retrospective Database Analysis checklist [28], which adapted the Strengthening the Reporting of Observational Studies in Epidemiology (STROBE) Statement: Guidelines for Reporting Observational Studies [29] for quality assessment of selected studies.

We assessed the included studies across the 22 criteria of the STROBE statement guidelines. On a three-level scale, we awarded 0 if the “criterion was not met”, 1 if the “criterion was partially met” and 2 “criterion was fully met”. When the criterion was not applicable to the article, it was marked as “NA”.

Maximum obtainable score across all criteria was 54 (100%). We converted the cumulative quality scores of each study to percentage quality scores. Using the 70% benchmark, we classified papers into high quality, if the study scored ≥ 70%, medium quality if the study scored from 50 to <70% and low quality if the study scored < 50%.

## Results

In this results section, we present a summary of search results, quality assessment results, distribution of studies that assess adolescent maternal health services in LMICs, characteristics of the included studies, and results of the thematic summary.

### Summary of search results

**Fig. 1:**
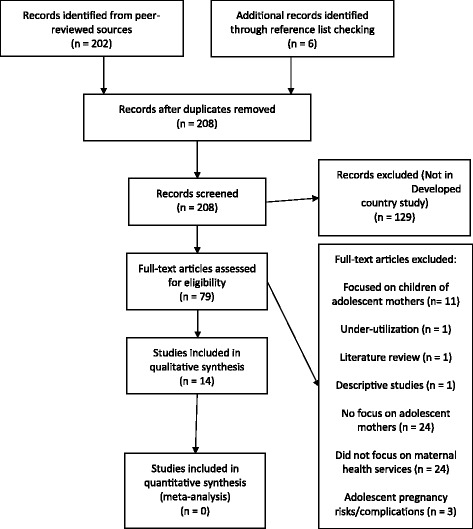
PRISMA diagram summarizing search process

Two hundred one records were retrieved after all duplicates were removed. After applying the set exclusion criteria, 14 articles remained that met our inclusion criteria for review [Fig. 1].

### Results of quality assessment

Six of the 14 studies were adjudged to be of high quality [30–35], and eight studies were adjudged to be of medium quality [36–43] [Additional file 1].

There were no significant inter-author(s) or inter-periodic differences. However, based on the quality framework that we applied [28], we observed that three main reasons (criteria) for lower quality scores were because author(s) did not “describe any efforts to address potential sources of bias”, “indicate number of participants with missing data for each variable of interest”, and/or, “discuss limitations of the study, taking into account sources of potential bias or imprecision”.

### Geographic distribution of assessments of adolescent MHS utilisation in LMICs

Additional file 2 is the completed data extraction sheet which shows that there have been 48 assessments of MHS utilisation by adolescent mothers conducted in 32 different countries and published within 14 different studies. The studies were conducted in Bangladesh, Benin, Bolivia, Brazil, Burkina Faso, Cambodia, Cameroon, Chad, Comoros, Ethiopia, Ghana, Guatemala, Guinea, India, Indonesia, Ivory Coast, Kenya, Madagascar, Malawi, Mali, Mozambique, Nepal, Nicaragua, Niger, Nigeria, Peru, Senegal, Tanzania, Togo, Uganda, Zambia and Zimbabwe [30–43]. There have been reassessments in some countries in later years following the first assessment including India [31, 41], Malawi [31, 33, 34], Mali [31, 42], Nepal [31, 32], Niger [33, 34], Nigeria [33, 40] and Uganda [31, 33] [Additional file 2].

**Fig. 2:**
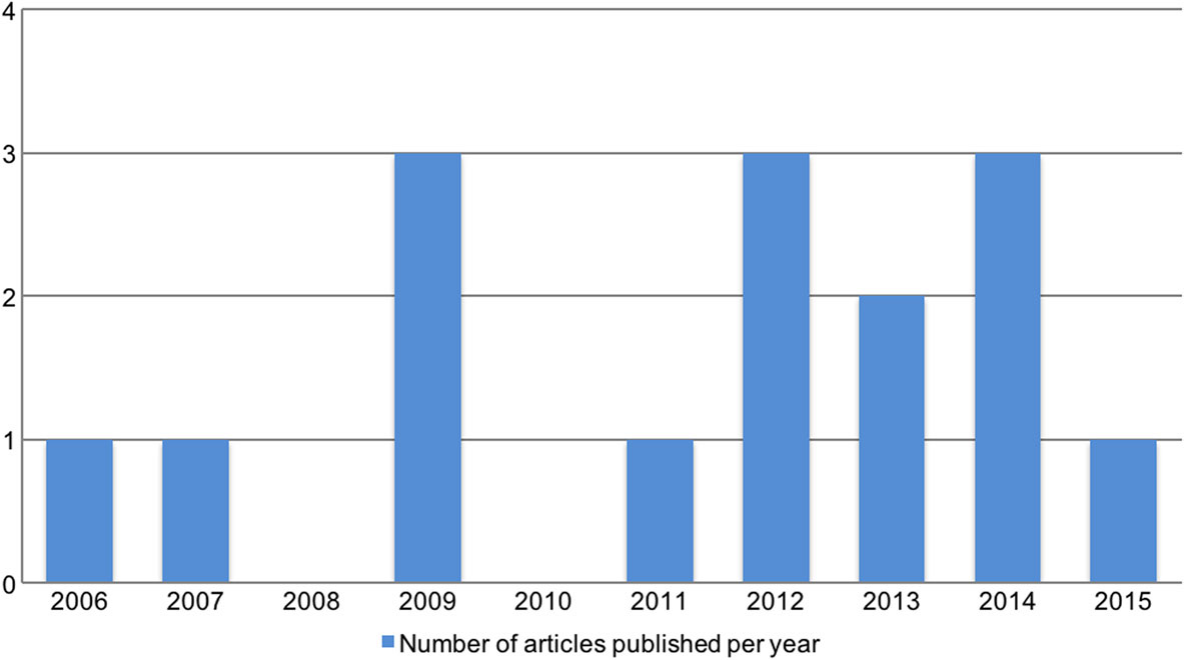
Time line of publication focused on adolescent MHS utilisation

The first study we retrieved which met our inclusion criteria was published in 2006 [31]. It was conducted in multiple countries [31]. Since then, between one and three studies have been published annually, except for 2008 and 2010 [Fig. 2].

### Characteristics of the included studies

All 14 studies used quantitative research approach in analysing MHS utilisation [30–43]. Almost all studies (13) sourced data for the assessment from secondary quantitative data, using either the Demographic Health Survey (DHS) series or National Family Health Survey (NFHS) (in two cases) [30, 31, 33–43] [Table 1]. The only study that collected primary quantitative data was conducted in Kathmandu, Nepal [32].

**Table 1:** Summary of characteristics of included studies

Characteristic	Number	Percentage
Data source	*n* = 14	
Primary data	1	7.1%
Secondary data	13	92.9%
Difference of year of publication to year of data source^a^	*n* = 48	
≤ 5 years	6	12.5%
> 5 years ≤10 years	38	79.2%
> 10 years	4	8.3%
Age group and focus	*n* = 14	
Adolescents alone (15–19)	10	71.4%
Comparative with other groups (15–19 vs. 20–24/20–34/35–49)	4	28.6%
Limited to married adolescents	*n* = 14	
Limited	10	71.4%
Included unmarried adolescents	4	28.6%
MHS studied	*n* = 14	
Delivery only	1	7.1%
ANC and delivery	6	42.9%
ANC, delivery, PNC	7	50.0%
Specific MHS characteristic studied	*n* = 14	
Full ANC (defined as Minimum 3 ANC visits, Tetanus Toxoid injection, Folic acid and Iron tablets), SBA present at delivery	1	7.1%
Full ANC (defined as Minimum 3 ANC visits, Tetanus Toxoid injection, Folic acid and Iron tablets), SBA present at delivery, Facility-based delivery, Skilled personnel provided PNC	1	7.1%
Full ANC (defined as Minimum 3 ANC visits, Tetanus Toxoid injection, Folic acid and Iron tablets), SBA present at delivery, Skilled personnel provided PNC	2	14.3%
Number of ANC visits (<4 - inadequate vs. 4 - adequate), SBA present at delivery	1	7.1%
Number of ANC visits (<4 - inadequate vs. 4 - adequate), SBA present at delivery, Skilled personnel provided PNC	3	21.4%
SBA present at delivery and Facility-based delivery	1	7.1%
Skilled personnel present for ANC at least one visit, SBA present at delivery	1	7.1%
Skilled personnel present for ANC, SBA present at delivery and Facility-based delivery	1	7.1%
Skilled personnel present for ANC, SBA present at delivery, Facility-based delivery and Skilled personnel provided PNC	1	7.1%
Skilled personnel provided ANC, Facility-based delivery	1	7.1%
Timing of first ANC visit, Number of ANC visits (<4 - inadequate vs. 4 - adequate), Facility-based delivery and SBA present at delivery	1	7.1%

^a^Numbers are based on country assessments (48) within the 14 studies

Of the 48 different country assessments, six used data that was 5 years old or less at the time of publication [31, 32, 35, 36, 38–41]. 37 country assessments used data 6 to 10 years old at the time of publication [30, 31, 33, 34, 37, 42, 43] and five country assessments used data that was over 10 years old already at the time of conduct [31, 33] [Table 1].

Seven studies reported on utilisation amongst adolescent mothers alone [30, 34–36, 38–43], one study compared utilisation amongst women aged 15–18 years and 19–23 years at the time of the survey with a birth in the previous 3 or 5 years [31]. One study reported on utilisation amongst mothers <20 years old and mothers 20–35 years [32] and another compared utilisation amongst age groups 15–19 years vs. 20–34 years vs. 35–49 years [33] [Table 1].

Six studies focused on adolescent mothers that were married [30, 32, 34, 35, 37, 38, 40, 42], while the other four were not specific to married adolescents [31, 33, 36, 39] [Table 1].

Six studies looked at MHS utilisation by adolescent mothers across the whole continuum of care (antenatal care (ANC), delivery and postnatal care (PNC)) [30, 35, 37, 38, 40, 42]. Six studies looked at ANC and delivery [31–34, 36, 41], one study assessed ANC and PNC [43] and one study assessed utilisation of delivery services only [39] [Table 1].

For ANC, specific characteristics of assessed services included presence of skilled personnel for ANC [31, 32, 36, 38], number of ANC visits [33, 34, 40, 42, 43], timing of first visit [33] and use of full ANC (defined as minimum 3 ANC visits, Tetanus Toxoid injection, folic acid and iron tablets) [30, 35, 37, 41] [Table 1].

For delivery, characteristic assessed were the presence of skilled birth attendant (SBA) at delivery [30, 31, 33–42] and facility-based delivery [32, 33, 36–39] [Table 1].

While for PNC, assessment focused on whether or not the care was provided by a skilled personnel [30, 35, 37, 38, 40, 42, 43] [Table 1].

### Findings of the thematic summary

We present our findings under three key themes: Factors considered by researchers in assessing adolescent MHS utilisation, factors assessed as statistically significant, and other findings on MHS utilisation reported in the literature.

### Factors considered by researchers in assessing adolescent MHS utilisation

**Fig. 3:**
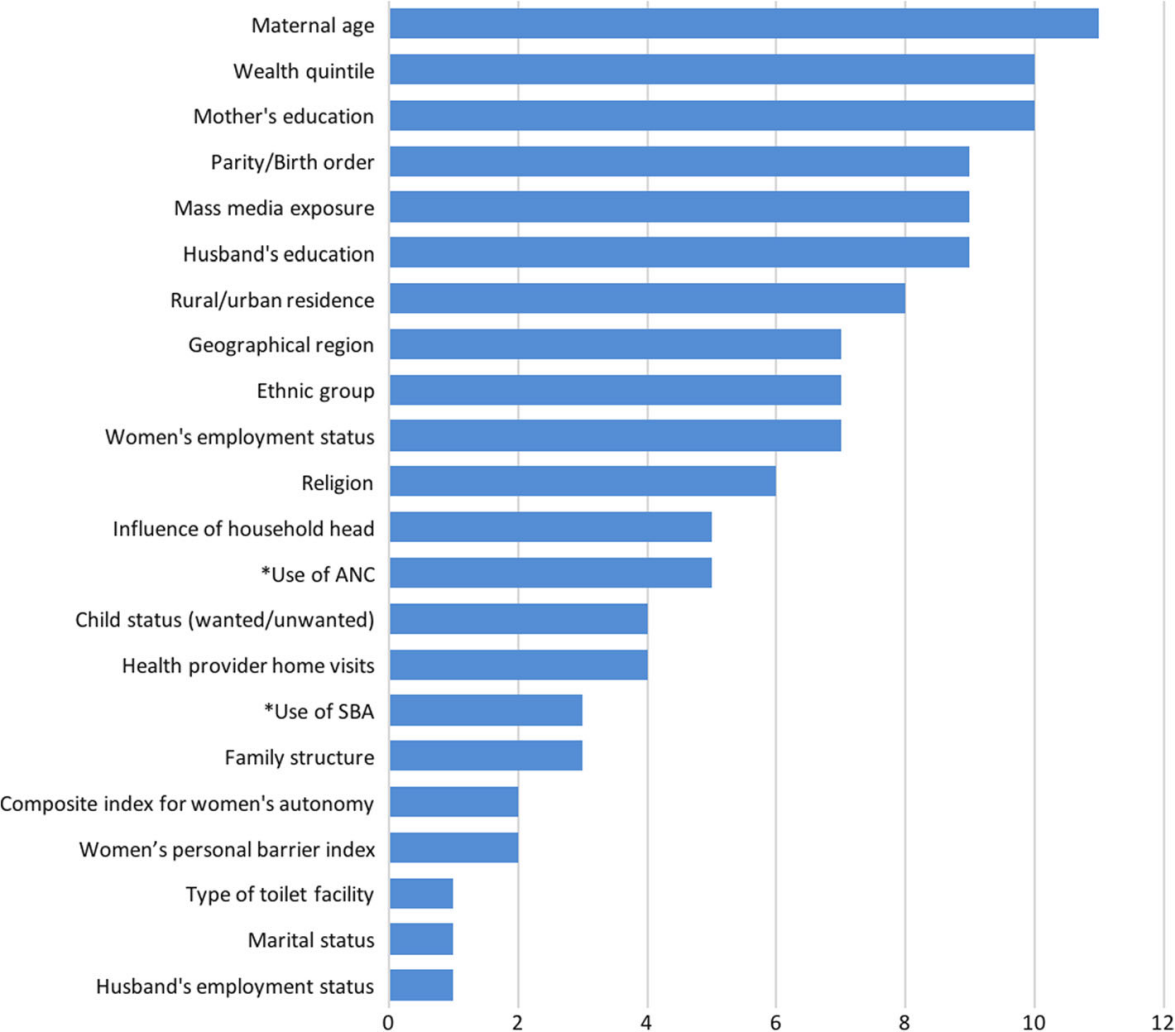
Predictor variables for assessing factors affecting adolescent MHS utilisation considered by researchers

Most commonly considered predictor variables were age of the mother (11 studies) [31, 33–38, 40–43], education status of mother (10) [30, 34–36, 38–43], wealth quintile (10) [30, 34–36, 38–43], education of the husband (9) [30, 34–36, 39–43], mass media exposure (9) [30, 34–36, 39–43], parity/birth order (9) [30, 34–36, 39–43], rural/urban residence (8) [34, 36, 38–43], employment status of the woman (7) [30, 34, 36, 38, 40, 42, 43], ethnic group (7) [30, 34, 35, 40–43], geographical region (7) [30, 35, 36, 38, 40–43], religion (7) [30, 35, 36, 38, 40, 41, 43], influence of household head (5) [32, 34, 40, 42, 43], health provider visits (4) [30, 35, 39, 41], and wanted/unwanted child status (4) [35, 39, 42, 43] [Fig. 3].

Less commonly considered predictor variables include family structure (3) [35, 38, 41], women’s personal barrier index (2) [42, 43], composite index for women’s autonomy (2) [34, 35], husband’s employment status (1) [36], and marital status (1) [30]. One study considered type of toilet at facility specifically for SBA (1) [39] [Fig. 3].

For predictor variables of SBA and PNC utilisation, six studies considered ANC utilisation [30, 34, 39, 40, 42, 43]. For PNC utilisation, three studies considered the use of SBA [30, 40, 42].

### Statistically significant factors influencing adolescent MHS utilisation

Excluding the two multi-country studies [31, 33] and the Kavitha et al. study in India [37] that focused on the influence of age on MHS utilisation, comparing adolescents with older women, the remaining 11 studies provide details on the most prevalent statistically significant predictor variables for adolescent MHS utilisation [30, 32, 34–36, 38–43].

Based on the significance level of *p* ≤ 0.05, Table 2 presents the frequency of the statistically significant predictor variables as well as their strength of association in predicting adolescent MHS utilisation. When analysed, factors such as education of the adolescent mother, husband’s education, wealth quintile, parity, region, family structure, child status (wanted/unwanted) and women’s personal barrier index were consistently highly statistically significant (*p* ≤ 0.01) [Table 2]. Similarly, use of ANC was highly statistically significant for the use of SBA while both uses of ANC and SBA were highly statistically significant for the use of PNC in all studies that reported the variable [Table 2]. Though statistically significant, the strength of significance was not as strong in all cases with predictor variables such as rural/urban residence, socio-ethnic group, religion, maternal age, women’s employment status, and health worker visit [Table 2].

**Table 2:**
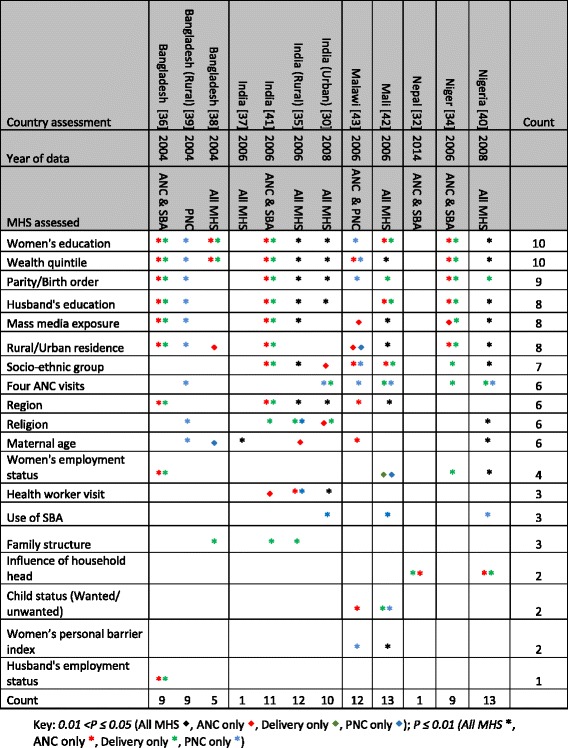
Distribution of predictor variables assessed to be significant in the literature with their estimated strengths of association

**Fig. 4:**
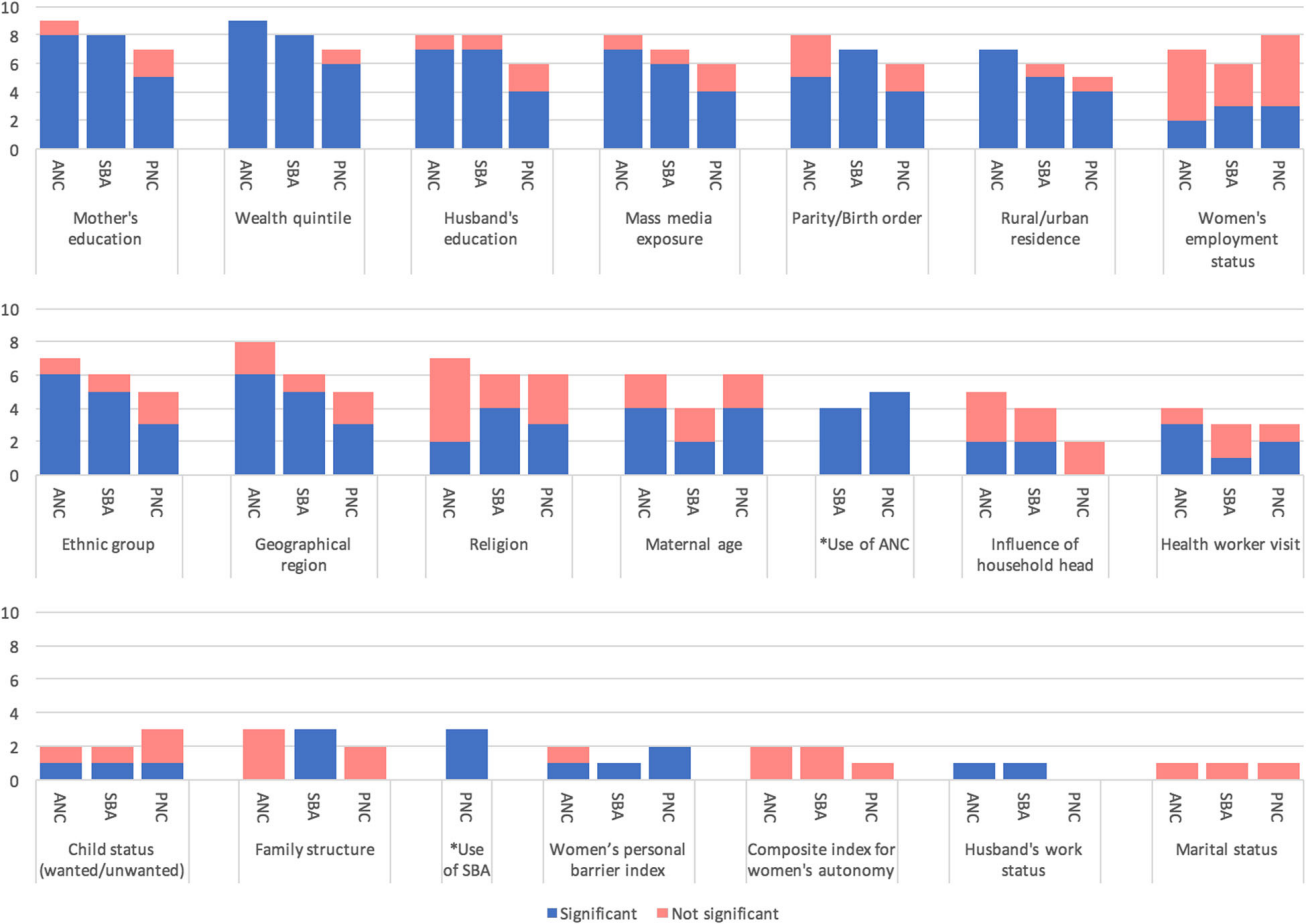
Number of statistically significant variables from studies that assessed the predictor variable for the different maternal health services

For ANC, wealth quintile was assessed as being statistically significant in all studies that assessed the variable [30, 34–36, 38–43], rural/urban residence (all seven studies) [34, 36, 38–43], education of the adolescent mother (eight of nine studies) [30, 34–36, 38, 40–42], husband's education (seven of eight studies) [30, 34–36, 40–43], and mass media exposure (seven of eight studies) [34–36, 39–43] [Fig. 4].

For SBA, wealth quintile was assessed as being statistically significant in all studies that assessed the variable (eight of eight studies) [30, 34–36, 38, 40–42], as well as in all studies that assessed education of the adolescent mother (eight of eight studies) [30, 34–36, 38, 40–42]. Similarly, all studies that assessed parity were statistically significant [30, 34–36, 40–42]. Mass media was statistically significant in six of seven studies [34–36, 40–42] while rural/urban residence was significant in five in six studies [34, 36, 40–42]. ANC utilisation was reported to be significant for SBA utilisation in all four studies that considered it as a predictor variable [30, 34, 40, 42] [Fig. 4].

For PNC, use of SBA was assessed as being statistically significant in all three studies that assessed the variable [30, 40, 42], wealth quintile (six of seven studies) [30, 35, 39, 40, 42, 43], adolescent mother’s education (five of seven studies) [30, 35, 39, 40, 43] and husband's education (four of six studies) [30, 35, 39, 40] [Fig. 4].

In all three studies conducted in India which tested multiple predictor variables [30, 35, 41], religion and health worker visit(s) were deemed a significant factor for MHS utilisation [Table 2]. Women’s employment status was not significant in any of the studies conducted in India [30, 35, 41].

### Other findings on maternal health services utilisation reported in literature

In the two comparative multi-country studies [31, 33], the evidence suggested that adolescents have lower MHS utilisation than older women with similar background characteristics. Specifically, adolescents were more likely to receive inadequate ANC and have unskilled birth attendance. There were also significant differences in the levels of MHS across countries. However, there was no evidence to suggest any significant variations across countries in the observed patterns of MHS utilisation by maternal age [33].

Only two studies, conducted in Nigeria and urban India reported percentage adolescent MHS utilisation [35, 40].

## Discussion

This systematic review mapped out the assessment of factors influencing adolescent MHS utilisation in LMICs, highlighting the distribution, quality and characteristics of studies that focus on this limited area of research. The review identified commonly used predictor variables in the assessment of adolescent MHS utilisation and predictor variables that have been shown to be significant, including the strength of their significance. The review also showed some evidence that there is poor utilisation by adolescent mothers compared to older mothers.

This review needs to be interpreted carefully, bearing in mind some of its limitations. Firstly, the search was limited to articles published in English language, as such, papers from developing Latin America and Francophone Africa countries may have been missed out. Secondly, the same group of researchers authored six out of the 14 included articles over a period of 3 years [34, 35, 40–43]. This similarity in author profile could affect the conclusions that we reach, because of the potential for the authors to make similar decisions and processes in the conduct of their research.

Despite overwhelming evidence suggesting that adolescents mothers are uniquely different from the general women’s population and that they are a particularly vulnerable and deprived population predisposed to worse maternal health outcomes compared to older age group women [6, 8–12, 44, 45], findings of our review show that there is limited number of studies published in the area of adolescent utilisation of critical MHS. Ten years since the first adolescent MHS utilisation study was published in 2006, it appears that there remains minimal interest in the topic. This becomes even more apparent when a comparison is made with the plethora of research that has been conducted on utilisation of MHS amongst older age group women [46–67]. The reason for the low focus on a vulnerable group like adolescent mothers is not particularly clear, but may not be unconnected to the inherent challenges in collecting data from this cohort. Firstly, data from demographic health surveys in several LMICs suggest that fertility rates amongst adolescents are lower than in women in their twenties and early thirties, as such the ‘chance’ of finding adolescent mothers for age-specific surveys are lower compared to mothers in their twenties [68–72]. Secondly, the issue of adolescents getting pregnant remain a culturally complex one in many LMICs [73] and as such capacity to survey sufficiently large numbers for sensible analysis may be further complicated, due to barriers such as lack of consent, shame of the adolescent mother for having a baby and her lack of power to have a conversation on such matters with a ‘stranger’ [73].

When studies have been published, our review points to the need to address some quality issues in under to improve reports on MHS utilisation assessments amongst adolescents. In line with best practices [28], authors need to ensure that they describe the management of bias, missing data and discuss limitations of their study. In addition, as these assessments mainly constitute observational studies, there is a need to highlight percentage utilisation data of adolescent MHS utilisation before presenting factors influencing utilisation. Only two studies did this in our review [35, 40].

All studies included in our study [30–43] used quantitative research methods in assessing MHS utilisation amongst adolescents. No study used qualitative research methods. Qualitative methods have been used extensively in healthcare [74, 75] and they offer a unique opportunity for researchers to be able to answer the “why” and not just the “what” [76]. Particularly as it relates to adolescents, there are several “why’s” that would need to be answered before effective strategies to improve their MHS utilisation can be implemented. In addition, qualitative methods may provide a more confidential platform for adolescents to discuss this sensitive topic. We believe that there is significant merit in supplementing survey-based approaches, using quantitative methods with qualitative methods for getting a better understanding of the challenges and other factors influencing adolescent mother’s care seeking patterns in different contexts. Use of such mixed methods approaches would provide the holistic perspective required for a broader understanding of adolescent MHS utilisation [77].

Only one study [32] collected primary evidence to assess MHS utilisation of adolescents. The remaining 13 studies [30, 31, 33–43] used different country-specific secondary data sources like the DHS. The DHS series are generally well renowned for their robustness and quality [78, 79]. However, there has to be some concern about the time lag between the date of publication of the DHS datasets and the date that researchers analyse them. This is particularly important especially if such analyses are to be relevant for ‘up-to-date’ policy-making. Four country assessments were based on data that was over 10 years old already at the time of analysis and 38 country assessments were based on data between 5 and 10 years old. The reality is that datasets for sub-set (adolescent population) analyses, like that of the DHS, are not immediately available following completion of the primary survey that generated the data. This may be the reason for the delay in subsequent secondary analyses. Following such delays, the relevance of findings from these secondary analyses may be called to question, specifically for adolescents, who continue to change from generation to generation, even in the space of 10 years. The needs, aspirations and characteristics of Generation X are different from Y and so are the needs of Generation Y entirely different from Generation Z [80, 81]. Similarly, the factors that influence MHS utilisation may be different amongst adolescents across generations. It appears that when such considerably wide time interval between dataset availability and analysis is the case, then the adolescents from whom the data had been collected are not the same for whom planning and policy choices are required.

From our findings, there also appears to be lots of focus on adolescent mothers who are already married [30, 32, 34, 35, 37, 40–43], ignoring the unmarried ones, who may be in even more precarious situations to be able to access MHS if they got pregnant [82]. This exclusion of unmarried adolescent mothers may in itself lead to some form of selection bias [83], thereby skewing results and affecting the interpretation of findings. The reason for the focus on married adolescent mothers is not too clear, but it may not be unconnected to possibly low numbers of unmarried adolescent mothers recruited in the primary surveys that were conducted to provide the datasets that the authors used for analysis. Secondly, some of the original surveys from which secondary analyses were subsequently conducted only collected data from within family settings that had married women [35, 37, 41, 42].

Excluding the multi-country studies, only two countries, Bangladesh [36, 38, 39] and India [37, 41] have had the same data source used for analysis on adolescent MHS utilisation multiple times. However, in both countries, the assessments used the same dataset for analysis, yet conclusions were not the same, regarding what factors were found to be significant. This, therefore, calls into question the quality of the analyses being done and highlights the need for more careful analysis and verification of findings. Also, we observed that even within the same countries, selection of predictor variables for consideration was not consistent. Our opinion is that selection of predictor variables for adolescent MHS utilisation must be based on the availability of reliable data, consideration for peculiarities of the specific setting and insight from literature focused on research conducted in similar settings.

With education of the adolescent mother being reported as statistically significant for MHS utilisation in all surveyed countries (except Malawi), there is a case for focusing on broader girl child education strategies. Similarly, education of the husband was reported to be significant in seven studies, and with the influence of the husband reported to be the most influential in making adolescent mothers use MHS [32], it is critical to include men to increase uptake of MHS by adolescents.

Findings from our review suggest that adolescent mothers are more likely to utilize MHS for their first pregnancy/delivery, but less likely to utilize MHS when they have more children [30, 34–36, 40–43]. There is, therefore, a need to make adolescents more aware of the additional risks that they face in pregnancy because of their ‘adolescence’. Our review suggests that there is an opportunity to leverage ANC attendance as a platform for advocacy to encourage and stimulate subsequent SBA utilisation by adolescents, especially as all five studies in our review that considered ANC utilisation as a predictor variable reported it as significant for SBA and PNC utilisation [30, 34, 39, 40, 42], which interphases with arguably the most critical period of the entire pregnancy for adolescents - delivery. The World Health Organization recommends that health care providers should be “seizing the opportunities” that patient engagements like ANC provide [84]. Evidence from the literature shows that ANC offers an opportunity to sensitize adolescent mothers about utilisation of MHS and promote healthy lifestyles that could potentially improve long-term health outcomes for them and their yet unborn child [19, 85]. For example, family planning counselling could be integrated into ANC, continued as part of PNC and this could potentially have a positive impact on the adolescent’s use of contraception after delivery. It is also a platform to implement a birth preparedness plan, ensuring that adolescent mothers can be better prepared for the birth itself including identifying the closest facility to manage them in the case of complications. However, this integration of services needs to be achieved, without overloading the already stretched workforce in many LMICs as well as providing an inclusive service for both married and unmarried adolescents [86].

Seven out of nine studies that looked at media exposure as a predictor variable, mass media exposure was found to be statistically significant. Going forward, with the proliferation of access to social media of young people globally, including in LMICs [87], ‘access to social media’ needs to be considered as a variable to be explored. We also opine that there is an opportunity to conduct research via electronic data collection, even via social media in order to target more adolescents, who otherwise will be uncomfortable talking to adults openly about their pregnancy etc. On the outcome side (MHS utilisation), while it is straightforward to report outcome measures such as attended ANC or not or attended PNC or not, there is need to capture indicators that describe the quality of care that adolescents also receive across the whole continuum of care. We note that four of the 14 included studies [30, 35, 37, 41], all conducted in India, actually reviewed whether adolescent mothers received Tetanus toxoid injection, folic acid and iron tablets. This is particularly important for adolescents because of their higher risk for poor maternal health outcomes. For them, it is not just about utilizing the services, but more about how well the services have been utilised.

No article was retrieved that assessed impact of intervention(s) in increasing MHS utilisation amongst adolescents. However, there have been many studies that reviewed the effectiveness of strategies in the wider women of reproductive age group, as evidenced in this recent systematic review [88]. More recently, another systematic review published in 2014, assessed the impact of user fees on MHS utilisation for all women [89]. To ensure that appropriate interventions are being properly targeted at increasing adolescent MHS utilisation, there is a need to build on the needed evidence to base decisions upon.

Even when broader age groups are being researched, it is critical to highlight adolescent mothers and conduct some form of subset analysis of adolescent mothers, because of their afore-described peculiarities. In our review, four studies did this [31–33, 37]. Such disaggregation of data is critical for planning and for better understanding and design of health systems. More recently, there have been global calls for presenting disaggregated data to ensure that inequities may be better highlighted [90], as may be the case with adolescent health MHS utilisation when compared to older women. In addition, such data may be able to support ‘business case’ development for the need to focus on adolescent MHS utilisation.

## Conclusions

Clearly, there are notable similarities between countries with regards to factors that affect adolescent MHS utilisation, especially maternal education and wealth index. Emphasis thus needs to be placed on educating girls and ensuring that financial barriers do not limit their access to critical care. However, there may be some context-specific factors in different countries, which need to be considered when designing interventions aimed at improving adolescent MHS utilisation. This study highlights the need for more robust evidence on how to achieve this. We need innovative approaches that incorporate both real-time quantitative and qualitative research methods in studying access, utilisation and quality of MHS for adolescent within specific settings. These studies should include ‘all adolescents’ and not the ‘easy to capture’ adolescents [18]. This will bridge the equity gap and promote universal health coverage.

Increasing access to and utilisation of quality MHS for adolescents especially in the 20 countries responsible for 82% of global adolescent maternal deaths [6], will contribute significantly to a reduction in maternal mortality. Efforts geared towards improving maternal health care for adolescents are consistent with the SDGs, which also focus on girl child education, preventing early pregnancy and removing financial barriers to care [91]. One thing we cannot afford to do again in the post-2015 era is to “leave them behind”.

## Additional files

Additional file 1: Quality assessment of included studies. Results of the quality assessment of the 14 included studies using the International Society for Pharmaco-economics and Outcomes Research (ISPOR) Good Research Practices for Retrospective Database Analysis checklist. (XLSX 31 kb)
Additional file 2: Completed data extraction sheet of systematic literature review. Full data extracted from the 14 included studies for the systematic literature review. (XLSX 31 kb)

## Abbreviations

ANC: Ante-Natal Care
DHS: Demographic Health Survey
ISPOR: International Society for Pharmaco-economics and Outcomes Research
LMICs: Low and middle income countries
MDGs: Millennium development goals
MHS: Maternal Health Service
NFHS: National Family Health Survey
PNC: Post-Natal Care
SBA: Skilled birth attendant
SDGs: Sustainable development goals
STROBE: Strengthening the Reporting of Observational Studies in Epidemiology
